# A Circadian Hygiene Education Initiative Covering the Pre-pandemic and Pandemic Period Resulted in Earlier Get-Up Times in Italian University Students: An Ecological Study

**DOI:** 10.3389/fnins.2022.848602

**Published:** 2022-04-14

**Authors:** Sara Montagnese, Lisa Zarantonello, Chiara Formentin, Gianluca Giusti, Chiara Mangini, Cheryl M. Isherwood, Paolo Ferrari, Antonio Paoli, Daniela Mapelli, Rosario Rizzuto, Stefano Toppo, Debra J. Skene, Roberto Vettor, Rodolfo Costa

**Affiliations:** ^1^Department of Medicine, University of Padova, Padua, Italy; ^2^Chronobiology Section, Faculty of Health and Medical Sciences, University of Surrey, Guildford, United Kingdom; ^3^reMedia Web Agency S.r.l., Padua, Italy; ^4^Department of Biomedical Sciences, University of Padova, Padua, Italy; ^5^Department of General Psychology, University of Padova, Padua, Italy; ^6^Department of Molecular Medicine, University of Padova, Padua, Italy; ^7^Department of Biology, University of Padova, Padua, Italy; ^8^Institute of Neuroscience, National Research Council (CNR), Padua, Italy

**Keywords:** circadian hygiene, chronotype, sleep, university students, academic performance, pandemic

## Abstract

The aims of the present study were to obtain sleep quality and sleep timing information in a group of university students and to evaluate the effects of a circadian hygiene education initiative. All students of the University of Padova (approximately 64,000) were contacted by e-mail (major campaigns in October 2019 and October 2020) and directed to an *ad hoc* website for collection of demographics and sleep quality/timing information. Participants (*n* = 5,740) received one of two sets of circadian hygiene advice (“A regular life” or “Bright days and dark nights”). Every month, they were then asked how easy it had been to comply and provided with the advice again. At any even month from joining, they completed the sleep quality/timing questionnaires again. Information on academic performance was obtained *post hoc*, together with representative samples of lecture (*n* = 5,972) and examination (*n* = 1,800) timings, plus lecture attendances (*n* = 25,302). Fifty-two percent of students had poor sleep quality, and 82% showed signs of social jetlag. Those who joined in October 2020, after several months of lockdown and distance learning, had better sleep quality, less social jetlag, and later sleep habits. Over approximately a year, the “Bright days and dark nights” advice resulted in significantly earlier get-up times compared with the “A regular life” advice. Similarly, it also resulted in a trend toward earlier midsleep (i.e., the midpoint, expressed as clock time, between sleep onset and sleep offset) and toward a decrease in the latency between wake-up and get-up time, with no impact on sleep duration. Significant changes in most sleep quality and sleep timing variables (i.e., fewer night awakenings, less social jetlag, and delayed sleep timing during lock-down) were observed in both advice groups over approximately a year, mostly in association with pandemic-related events characterizing 2020. Early chronotype students had better academic performances compared with their later chronotype counterparts. In a multivariate model, sleep quality, chronotype and study subject (science and technology, health and medical, or social and humanities) were independent predictors of academic performance. Taken together, these results underlie the importance of designing circadian-friendly university timetables.

## Introduction

Chronotype reflects an individual’s natural inclination to place his/her activity/sleep within different intervals of the 24-h day and is partly genetically determined (Ashbrook et al., 2020). Children tend toward earlier, morning chronotypes, whereas adolescents and young adults exhibit a sharp change toward eveningness (which is more pronounced/prolonged in males), but then mature adults/older individuals become progressively more morning-like (Roenneberg et al., 2004). Late chronotype individuals are penalized by the Western “social clock,” which forces them to study/work/function during the early part of the day, counter to their natural biological clock (Gentry et al., 2021). It has been reported that early classes/early school tests in evening-type adolescents adversely affect their academic performance, especially in scientific subjects (Zerbini et al., 2017) and that changes in school start times may help improve sleep (Winnebeck et al., 2020; Meltzer et al., 2021) and, possibly, also grades (Zerbini et al., 2017). Less but similar information is available on the relationship between study timetables, sleep timing/length, and academic performance in university students (Beşoluk et al., 2011; Li et al., 2018; Smarr and Schirmer, 2018). In addition, there are also studies on the effects of sleep deprivation on university students’ athletic performance and wellbeing (Hodge et al., 2012; Bolin, 2019). A number of different initiatives/courses or interventions directed at either general or specific university students’ populations (Tsai and Li, 2004; Brown et al., 2006; Trockel et al., 2011; Quan et al., 2013; Kloss et al., 2014; Ye and Smith, 2015; Levenson et al., 2016; Hershner and O’Brien, 2018) have shown encouraging results either in terms of increased sleep hygiene literacy (Tsai and Li, 2004; Brown et al., 2006; Quan et al., 2013; Kloss et al., 2014; Ye and Smith, 2015; Hershner and O’Brien, 2018) or actual improvement in sleep habits and/or sleep quality (Brown et al., 2006; Trockel et al., 2011; Levenson et al., 2016; Hershner and O’Brien, 2018).

The aims of this study were to obtain comprehensive sleep quality/timing information and to evaluate the effects of a university welfare-based circadian hygiene initiative—directed at all students—on sleep and academic performance. As the study progressed, it became apparent that novel information could also be obtained on the effects of the pandemic-related lockdown on sleep–wake behavior.

## Materials and Methods

### Overview of the Survey and Educational Initiative

**FIGURE 1:**
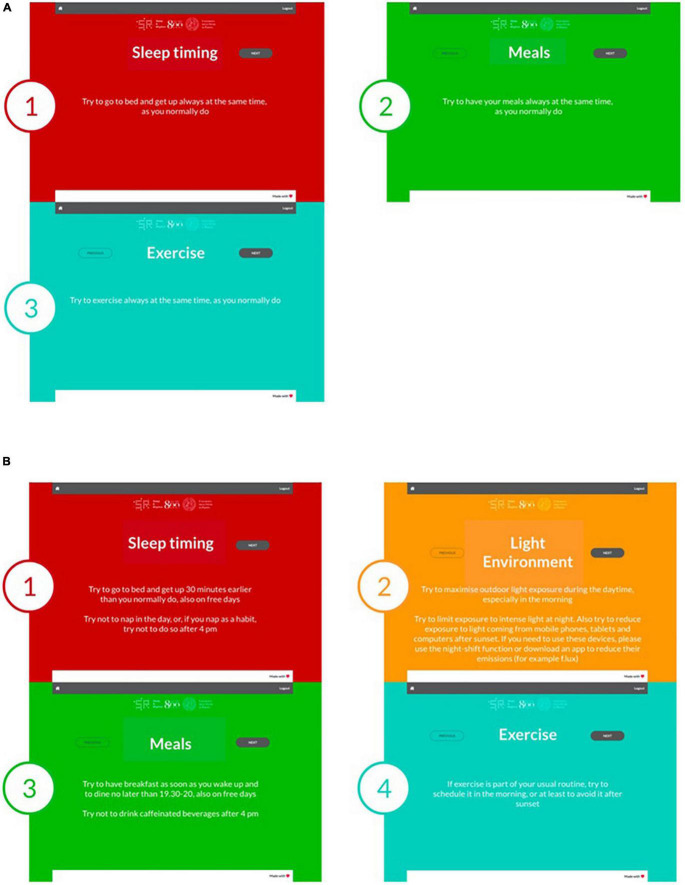
Circadian hygiene advice. Advice provided in the “A regular life” **(A)** and the “Bright days and dark nights” **(B)** groups. The numbering of each panel refers to the temporal sequence in which the advice was provided (on subsequent smartphone/tablet/computer screens).

An *ad hoc* full-responsive website was created for collection of the relevant information, with either an Italian or English interface to choose from. All active students at the University of Padova (approximately 64,000) were contacted by the university welfare offices on October 28, 2019, with an e-mail message including a brief description of the initiative and its aims, an invitation to participate, and a “call to action” button leading to the website.^1^ This could only be accessed by use of the personal Shibboleth University of Padova username/password. Students who joined the initiative went on to receive reminders on the 28th of each subsequent month to access the website again to provide additional information and receive advice. The website was always open for newcomers to join; access after each reminder was possible for 7 days. On joining for the first time, students were asked to provide information on demographics, sleep–wake timing, night sleep quality, and diurnal sleepiness. They then received one of two sets of circadian hygiene advice (“A regular life” or “Bright days and dark nights,” Figure 1). Every month after joining, they were asked how easy it had been to comply with the advice and were provided with the advice again. At every even month from joining, they were asked to complete demographic, sleep–wake timing, night sleep quality, and diurnal sleepiness information again. They were also asked how easy it had been to comply, and finally, they were provided with the advice again (Supplementary Table 1). An additional information/recruitment campaign was run on October 28, 2020, which excluded any student who had already joined. Information on the participating students’ career parameters was obtained from the career office *post hoc*. Representative samples of the timings of electronic room bookings for lectures/examinations and of recorded, anonymous lecture attendances were also obtained *post hoc*.

### Demographics

Date and time of both first accessing and completing all procedures scheduled at time 0 (T0) were obtained. In addition to basic demographics, students also provided information on their height, weight, any problems with eyesight, any disease, and/or any medication they might be on. In relation to eyesight, diseases, and medication, a yes/no answer was a requirement in order to proceed. By contrast, the field to provide additional details on any of these three items could be left empty. Authors SM, CF, and CM, who are all practicing clinicians, independently classified listed eyesight problems, diseases, and drugs as worth considering or negligible; where their classifications differed, an agreement was sought for and reached.

### Sleep–Wake Profile

Carefully developed, user-friendly electronic versions with equal resolution and ease of completion on mobile phones, tablets, and computers of the following questionnaires were then presented on the full-responsive website.

#### The Sleep Timing Sleep Quality Screening (STSQS) Questionnaire

This provides a simple, overall assessment of sleep quality rated on a 0–10 visual analog scale (0 = worst, 10 = best sleep ever) and allows collection of information on habitual sleep timing (i.e., bed time, try to sleep time, sleep latency, night awakenings, wake-up and get-up time, Montagnese et al., 2009).

#### The Pittsburgh Sleep Quality Index (PSQI)

Responses to 19 questions are used to generate seven components (subjective sleep quality, sleep latency, sleep duration, sleep efficiency, sleep disturbance, use of sleep medication, daytime dysfunction), each of which is scored from 0 (best) to 3 (worst). These component scores are then summated to provide the total PSQI score (range = 0–21); scores of > 5 identify “poor sleepers” (Buysse et al., 1989; Curcio et al., 2013).

#### The Epworth Sleepiness Scale (ESS)

Subjects rate their likelihood of “dozing off” in eight different daytime situations, on a scale of 0 (unlikely) to 3 (very likely). The component scores are summated to provide a total score (range = 0–24); a score of ≥ 11 is considered abnormal (Johns, 1991; Vignatelli et al., 2003).

#### The Self-Morningness/Eveningness Self-ME Question

This is a validated, single-question assessment of chronotype through which participants qualify themselves as definitely morning, morning, evening, or definitely evening types (Turco et al., 2015).

#### The Ultrashort Version of the Munich ChronoType Questionnaire μMCTQ

This is an adaptation of the MCTQ (Roenneberg et al., 2003) from 17 to 6 essential questions, allowing for a quick assessment of midsleep (i.e., the midpoint, expressed as clock time, between sleep onset and sleep offset), social jetlag (in this instance, the uncorrected difference between midsleep on free and work/study days), and sleep duration (Ghotbi et al., 2020). Social jetlag was both treated as a continuum variable and also qualified as *positive* (difference between midsleep on free and work/study days > + 20 min), *absent* (−20 min < difference < + 20 min) and *negative* (difference < −20 min) (Korman et al., 2020).

### Circadian Hygiene Advice and Compliance

Two alternative sets of advice (novel in their formulation but grounded in circadian practice; Abbott et al., 2015) were provided (and remained the same on every subsequent “refresh” monthly reminder) with comparable expected benefits (i.e., balancing out because the first type of advice was slightly less prescriptive but probably easier to follow, and the second slightly more prescriptive but probably more difficult to follow) exerted *via* two different forms of habits’ adjustment (Facer-Childs et al., 2019). Both included elements of the recommendations routinely provided to patients with either a formal diagnosis and/or features of delayed sleep phase type (Abbott et al., 2015), which is common in adolescents and young adults.

**“A regular life,”** encouraging participants to regularize habits in relation to sleep–wake timing, meals, and exercise (Figure 1A). This intervention did not include advice on light exposure, as whereas the idea of sleep, meals, and exercise at regular times is extremely intuitive and easily interpreted, that of “light at regular times” seems less so. Therefore, when planning the intervention, we reasoned that advice on “light at regular times” would be prone to misinterpretation.

**“Bright days and dark nights,”** encouraging participants to advance their sleep–wake, meals, and exercise timing and to maximize/minimize light exposure in the first/second part of the day, respectively (Figure 1B).

The user-friendly, short format of the interventions was chosen to maximize the likelihood of them being read and considered by young, healthy individuals on a monthly basis and most likely on the screen of their mobile phone. Along the same lines, we did not modulate advice in relation to photoperiod or daylight saving time (DST), deliberately opting for repeated, identical advice, which could be read, reread (on refresh), and memorized easily, hopefully becoming part of the students’ routine.

This being a university-based welfare initiative, all students were provided with advice; that is, none served as placebo.

Participants subjectively rated their **compliance** with each piece of advice received on a 0 (never) to 10 (all the time) visual analog scale.

### Academic Performance

Information was obtained from the student career offices about each student’s university course, course year, total number of examinations passed, total number of “credits” (i.e., packages of formal lectures/practicals of variable length, depending on study subjects), and average marks (0–30, 18 = pass) at fixed dates of the year, on a 3-monthly interval (October 28, 2019; January 28, 2020; April 28, 2020; July 28, 2020 and October 28, 2020). These data were then matched with the “join the initiative” date and organized as baseline (T0) and subsequent time intervals. Studies were classed as health and medical (M), science and technology (S), and social and humanities (H), based on the Italian Ministry of University and Research reference tables. Courses have variable duration: most have a 3- plus 2-year structure (in this instance, students who went on to attend their master’s degree were qualified as fourth or fifth year), whereas a minority have a 4–6-year duration. Based on the information they provided and on the University of Padova criteria, students were classified as on-site, commuters, or off-site (i.e., non-compulsory lectures and/or registered for examinations only).

Significant differences in sleep–wake and other parameters were observed between students pursuing M, S, and H studies, which were thought to depend, to some extent, on lectures timing/duration and/or examinations timing. Thus, a sample of electronic room bookings for lectures (*n* = 5,972) was obtained *post hoc* on a representative week (November 4–10, 2019, i.e., close to study start; lectures were arbitrarily qualified as *morning/afternoon* if they started prior to/later than 13:00 hh:mm, respectively) and a sample of anonymous students’ recorded attendances (electronic swipe in–swipe out, *n* = 25,302; multiple swipe ins–swipe outs, *n* = 7,765) on October 30, 2020. This date was chosen because, due to novel, pandemic-related rules, all students were asked to swipe in–swipe out electronically. Finally, a sample of examination booking times was obtained, on a representative week [September 9–13, 2019 (*n* = 1,800 bookings)]; again, examinations were arbitrarily qualified as *morning/afternoon* if they started prior to/later than 13:00 hh:mm, respectively.

### Study Approval

The initiative was approved by the University of Padova (July 16, 2019, board meeting). Students were asked to accept/tick a general data protection regulation (GDPR)–compliant informed consent, including the wording “data may be used anonymously, in aggregate fashion for scientific purposes.” The study plan was then approved by the local ethics committee (4948/AO/20–AOP1939).

### Statistical Analysis

Descriptive results are expressed as mean ± SD/SE or as count/percentage. Normality was tested for by the Shapiro–Wilk test. Differences between normally distributed variables were examined by Student *t*-test/one-way analysis of variance (ANOVA; *post hoc*: Scheffé test). Differences between non-normally distributed variables were examined by Mann–Whitney *U*-test/Kruskal–Wallis ANOVA (*post hoc*: median test). Bonferroni correction for multiple comparisons was applied as appropriate. The effects of the circadian hygiene initiative and academic performance over time were analyzed by repeated-measures ANOVA (no missing data imputation procedures applied); η*_*p*_*^2^ was utilized as an indicator of the effect size. Factorial ANOVA was utilized for multiple grouping factors. A linear regression model was utilized to identify independent predictors of academic performance. The assumption behind the choice of variables for the linear regression was that they were either expected (age) or shown to affect sleep and/or academic performance. These were age, sex, study subject, commuting, sleep quality (continuous total PSQI score), and chronotype. Diagnostics were conducted as follows; normality: inspection of the histogram and the quantile–quantile plot of the residual; homogeneity of variance (homoscedasticity): inspection of the plot of residuals vs. predicted values; outliers: inspection of residual vs. predicted values and residuals vs. predictors, the leverage and residual plot, evaluation of the Cook Di (> 4/n); collinearity: correlation among the predictors and VIF > 3; and independence: inspection of the plot of residuals vs. predicted values. Interaction was tested by adding the predictor terms X1*X2, X1*X3, X2*X3, and so on, and detected between age and PSQI. Age was treated as a confounder. Analyses were carried out with Statistica, version 13.1 (Dell, Round Rock, TX, United States) and subsequently with version 14.0.0.15 (TIBCO, Palo Alto, CA, United States).

## Results

On December 19, 2020, 5,740 students (38% males; age 23 ± 5 years) had completed the first full set of questions (Supplementary Table 1); 55% had responded to the October 2019 call, 29% to the October 2020 call, and 16% had joined at different times between October 2019 and December 2020. Seven hundred forty students (31% males) attended health and medical (M), 2,439 (58% males) science and technology (S), and 2,492 (22% males) social and humanities (H) studies; 69 attended single courses. There were 2,981 students assigned to the “A regular life” and 2,759 to the “Bright days and dark nights” advice group; 1,144 students had one/more diseases, and 1,359 were on medication, with significant overlap (χ^2^ = 869, *p* < 0.0001); 1,295 reported minor eyesight issues.

### Sleep–Wake Profiles

There were 2,972 students (52%) who had an abnormal PSQI, with the most heavily affected components being subjective sleep quality, sleep latency, sleep disturbance, and daytime dysfunction; 714 (12%) had an abnormal ESS. Based on the Self-ME, 570 students qualified themselves as definitely morning, 1,896 as morning, 2,233 as evening, and 1,041 as definitely evening. As expected, the Self-ME classification was reflected in the more specific sleep timing variables obtained from the STSQS questionnaire. In addition, definitely evening/evening types had significantly worse PSQI scores compared with their more morning counterparts [6.9 ± 3.2/6.4 ± 3.0 vs. 5.8 ± 2.9/5.8 ± 3.0; *F*_(3, 5,736)_ = 34, *p* < 0.0001; all *post hoc* significant for definitely evening/evening vs. definitely morning and morning types]. Based on the μMCTQ, 240 students had negative, 755 no, and 4,703 positive social jetlag; 42 did not provide sufficient information. Average midsleep on study and free days was 03:54 ± 01:06 and 05:06 ± 01:12 (hh:mm), respectively. Average sleep duration on study and free days was 7.5 ± 1.2 and 8.4 ± 1.2 h, respectively.

Female students had earlier sleep–wake timing habits, more night awakenings, worse sleep quality, and more daytime sleepiness compared with male students (Table 1). In addition, they had earlier midsleep on both study and free days, and they more commonly classified themselves as morning chronotypes. Finally, their sleep duration was longer on free days (Table 1). Commuters had earlier sleep–wake timing habits [i.e., bed time: 23:24 ± 01:00 vs. 23:30 ± 01:00 hh:mm; *t*_(4,144)_ = 4.8; *p* < 0.001] compared with on-site students. Students attending H were older than those attending M, who, in turn, were older than those attending S studies (Table 2). Students attending M had earlier sleep–wake timing compared with students attending S/H studies. Finally, students attending H had worse sleep quality than those attending M/S studies (Table 2).

**TABLE 1:** Sleep–wake indices (mean ± SD) on joining the initiative at T0, by sex.

Questionnaire	Variable	Females (*n* = 3,566)	Males (*n* = 2,174)
STSQS	Bed time (hh:mm)	23:18 ± 00:54	23:42 ± 01:06*
	Try to sleep time (hh:mm)	23:51 ± 00:57	24:06 ± 01:04*
	Sleep latency (min)	24 ± 24	20 ± 18*
	Calculated sleep onset time (hh:mm)	24:15 ± 01:06	24:26 ± 01:09*
	Night awakenings (n)	1.0 ± 1.2	0.8 ± 1.0*
	Wake-up time (hh:mm)	07:34 ± 01:09	07:39 ± 01:15
	Get-up time (hh:mm)	07:56 ± 01:16	08:00 ± 01:21
	Latency between wake-up and get-up time (min)	21 ± 28	20 ± 31
PSQI	Total score (0–21)	6.5 ± 3.2	5.8 ± 2*
ESS	Total score (0–24)	6.7 ± 3.6	5.7 ± 3.3*
Self-ME	Definitely morning/morning/evening/ definitely evening (%)	11/34/38/16	8/31/40/21*
μMCTQ	Sleep onset study days (hh:mm)	24:03 ± 01:14	24:15 ± 01:15*
	Sleep offset study days (hh:mm)	07:34 ± 01:17	07:42 ± 01:21*
	Sleep duration study days (hours)	7.5 ± 1.2	7.4 ± 1.1
	Midsleep study days (hh:mm)	03:49 ± 01:05	04:00 ± 01:09*
	Sleep onset free days (hh:mm)	24:27 ± 01:15	01:06 ± 01:20*
	Sleep offset free days (hh:mm)	09:14 ± 01:21	09:24 ± 01:29*
	Sleep duration free days (hours)	8.4 ± 1.2	8.3 ± 1.1*
	Midsleep free days (hh:mm)	05:00 ± 01:09	05:15 ± 01:16*
	Social jetlag (hours)	1.2 ± 1.3	1.3 ± 1.4

*p < 0.001 (Bonferroni-corrected threshold: 0.05/20 = 0.0025).

*STSQS, Sleep Timing Sleep Quality Screening questionnaire; PSQI, Pittsburgh Sleep Quality Index; ESS, Epworth Sleepiness Scale; Self-ME, self-morningness/eveningness questionnaire; μMCTQ, ultrashort Munich ChronoType Questionnaire.*

**TABLE 2:** Sleep–wake indices [mean ± SE (95% CI)] on joining the initiative at T0, by study subject.

Questionnaire	Variable	Social and humanities (H; *n* = 2,492)	Health and medical (M; *n* = 740)	Science and technology (S; *n* = 2,439)
Age (years)		24.0 ± 0.1 (23.8–24.2)	23.2 ± 0.2*** (22.8–23.5)	22.4 ± 0.1 ^###,°⁣°°^ (22.2–22.6)
STSQS	Bed time (hh:mm)	23:26 ± 00:01 (23:24–23:29)	23:19 ± 00:02 *** (23:15–23:24)	23:27 ± 00:01° (23:25–23:30)
	Try to sleep time (hh:mm)	24:00 ± 00:01 (23:58–24:03)	23:46 ± 00:02 *** (23:41–23:50)	23:56 ± 00:01 ^#,°⁣°°^ (23:53–23:58)
	Sleep latency (min)	24 ± 0 (24–25)	21 ± 1 * (20–23)	21 ± 0 ^###^ (20–22)
	Calculated sleep onset time (hh:mm)	24:25 ± 00:01 (24:22–24:27)	24:07 ± 00:02 *** (24:02–24:12)	24:17 ± 00:01 ^###,°⁣°°^ (24:14–24:19)
	Night awakenings (n)	1.06 ± 0.02 (1.01–1.10)	0.82 ± 0.04 *** (0.73–0.90)	0.83 ± 0.02 ^###^ (0.78–0.87)
	Wake-up time (hh:mm)	07:45 ± 00:01 (07:42–07:48)	07:22 ± 00:02 *** (07:17–07:27)	07:31 ± 00:01 ^###,°^ (07:28–07:34)
	Get-up time (hh:mm)	08:08 ± 00:01 (08:05–08:11)	07:41 ± 00:02 *** (07:35–07:46)	07:51 ± 00:01 ^###,°⁣°^ (07:48–07:54)
	Latency between wake-up and get-up time (min)	23 ± 0 (22–24)	18 ± 1 *** (16–20)	20 ± 0 ^###^ (18–21)
PSQI	Total score (0–21)	6.6 ± 0.1 (6.5–6.7)	5.9 ± 0.1 *** (5.7–6.2)	6.0 ± 0.1 ^###^ (5.9–6.1)
ESS	Total score (0–24)	6.3 ± 0.1 (6.2–6.5)	6.5 ± 0.1 (6.2–6.7)	6.2 ± 0.1 (6.0–6.3)
Self-ME	Definitely morning/morning/evening/definitely evening (%)	10/33/38/19	11/33/41/15	9/33/39/19
μMCTQ	Sleep onset study days (hh:mm)	24:12 ± 00:01 (24:09–24:15)	23:55 ± 00:02 *** (23:50–24:00)	24:07 ± 00:01°^°⁣°^ (24:04–24:10)
	Sleep offset study days (hh:mm)	07:45 ± 00:01 (07:42–07:48)	07:19 ± 00:02 *** (07:13–07:24)	07:35 ± 00:01 ^###,°⁣°°^ (07:32–07:38)
	Sleep duration study days (hours)	7.56 ± 0.02 (7.51–7.60)	7.40 ± 0.04 ** (7.32–7.49)	7.45 ± 0.02 ^##^ (7.41–7.50)
	Midsleep study days (hh:mm)	03:58 ± 00:01 (03:56–04:01)	03:37 ± 00:02 *** (03:32–03:41)	03:51 ± 00:01 ^###,°⁣°°^ (03:48–03:53)
	Sleep onset free days (hh:mm)	24:55 ± 00:01 (24:52–24:58)	24:47 ± 00:02 (24:42–24:53)	24:55 ± 00:01 (24:52–24:58)
	Sleep offset free days (hh:mm)	09:18 ± 00:01 (09:15–09:21)	09:11 ± 00:03 (09:05–09:17)	09:20 ± 00:01 (09:16–09:23)
	Sleep duration free days (hours)	8.38 ± 0.02 (8.34–8.43)	8.40 ± 0.04 (8.31–8.49)	8.40 ± 0.02 (8.36–8.45)
	Midsleep free days (hh:mm)	05:06 ± 00:01 (05:04–05:09)	04:59 ± 00:02 (04:54–05:04)	05:07 ± 00:01° (05:05–05:10)
	Social jetlag (hours)	1.16 ± 0.03 (1.11–1.21)	1.42 ± 0.05 * (1.32–1.51)	1.29 ± 0.03° (1.24–1.34)

***p < 0.001. **p < 0.01 H. *p < 0.05. vs. M. ^###^p < 0.001. ^##^p < 0.01. ^#^p < 0.05 H vs. S. ^°⁣°⁣°^p < 0.001. ^°⁣°^p < 0.01. °p < 0.05 M vs. S. STSQS, Sleep Timing Sleep Quality Screening questionnaire; PSQI, Pittsburgh Sleep Quality Index; ESS, Epworth Sleepiness Scale; Self-ME, self-morningness/eveningness questionnaire; μMCTQ, ultrashort Munich ChronoType Questionnaire.

Students with diseases had more night awakenings [1.1 ± 1.2 vs. 0.9 ± 1.1; *t*_(5,737)_ = −5.0, *p* < 0.0001] and worse sleep quality [PSQI: 7.0 ± 3.5 vs. 6.1 ± 2.9; *t*_(5,737)_ = −8.6, *p* < 0.0001]; the same applied to students on medication.

Students who joined in October 2020 (during the pandemic; *n* = 1,697) had later sleep–wake timing [i.e., wake-up time: 07:48 ± 01:12 vs. 07:24 ± 01:06 hh:mm; *t*_(4,825)_ = −9.9, *p* < 0.001], less social jetlag [1.2 ± 1.7 vs. 1.3 ± 1.1 h; *t*_(4,799)_ = 3.9, *p* < 0.001], longer study days sleep duration [7.6 ± 1.2 vs. 7.4 ± 1.1 h; *t*_(4,808)_ = −6.3, *p* < 0.001], better sleep quality (PSQI abnormal 49% vs. 52%: χ^2^ = 4.6, *p* < 0.05), and less daytime sleepiness (ESS abnormal 10% vs. 14%: χ^2^ = 14.9, *p* < 0.001) compared with the pre-pandemic October 2019 cohort (*n* = 3,137). Of note, the two cohorts were comparable in age and female-to-male ratio, and any students who joined in between or later were excluded for purposes of this specific comparison.

### Circadian Hygiene Intervention and Self-Reported Compliance

**FIGURE 2:**
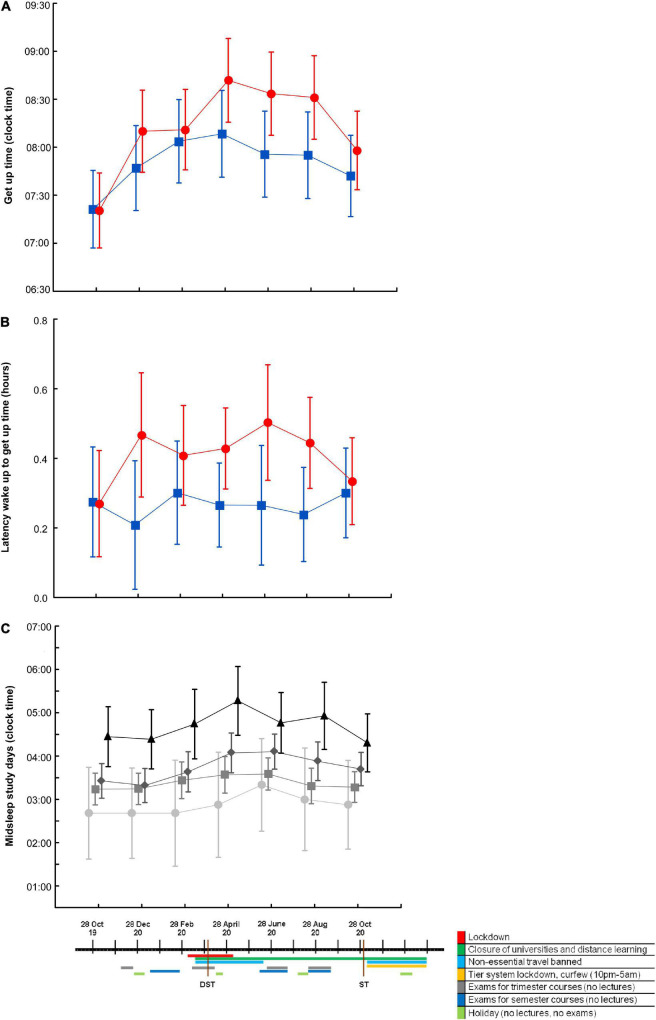
Sleep timing indices by circadian hygiene education group and by chronotype, over time and in relation to relevant 2020 events. Get-up time **(A)** and latency between wake-up and get-up time **(B)** in the “A regular life” (red circles; *n* = 30 at each time point) and the “Bright days and dark nights” (blue squares; *n* = 28 at each time point) groups. Differences were significant up to August 2020, that is T10 [get-up time: *F*_(5,365)_ = 17.0, *p* time < 0.0001 (η*_*p*_*^2^ = 0.189), *F*_(1, 73)_ = 4.2, *p* intervention = 0.042 (η*_*p*_*^2^ = 0.055), *F*_(5,365)_ = 1.2, *p* interaction n.s. (η*_*p*_*^2^ = n.s.); latency: *F*_(5,365)_ = 2.1, *p* time = 0.06 (η*_*p*_*^2^ = n.s.), *F*_(1, 73)_ = 5.9, *p* intervention = 0.02 (η*_*p*_*^2^ = 0.075), *F*_(5,365)_ = 1.7, *p* interaction n.s. (η*_*p*_*^2^ = n.s.)] and were abolished/reduced when October 2020 (T12), which coincided with the week after the transition from daylight saving time (DST) to standard time (ST), was included [get-up time: *F*_(6,336)_ = 11.9, *p* time < 0.0001, *F*_(1, 56)_ = 2.4, *p* intervention n.s.; *F*_(6,336)_ = 1.5, *p* interaction n.s.; latency: *F*_(6,336)_ = 0.9, *p* time n.s., *F*_(1, 56)_ = 3.1, *p* intervention = 0.08; *F*_(6,336)_ = 2.0, *p* interaction n.s.]. Midsleep on study days **(C)** in definitely morning (light gray circles), morning (gray squares), evening (dark gray diamonds), and definitely evening (black triangles) chronotypes [*F*_(3,345)_ = 5.3, *p* time < 0.001, *F*_(3, 69)_ = 4.0, *p* chronotype < 0.005, *F*_(15, 345)_ = 1.2, *p* interaction n.s., at T10; *F*_(6,312)_ = 4.7, *p* time < 0.001, *F*_(3, 52)_ = 5.6, *p* chronotype < 0.005, *F*_(18, 312)_ = 0.9, *p* interaction n.s., at T12]. Values are expressed as means ± 95% confidence intervals. n.s., not statistically significant.

Questionnaire completion rates decreased over time (Supplementary Table 1). While several hundred students completed the questionnaires at any given T (Supplementary Table 1), a total of 30 completed all even study times up to T12 in the “A regular life” group, and 28 in the “Bright days and dark nights” group. The two groups were comparable for sleep quality/timing, and female-to-male, study subject, and chronotype ratios. Significant differences between the two intervention groups were observed in get-up time and in the latency between wake-up and get-up time until T10 (earlier get-up time and shorter latency in the “Bright days and dark nights” group). Statistical significance was lost when T12, which coincided with the transition from DST to standard time (ST), was included (Figures 2A,B). At this moment, it was the behavior of the “A regular life” group that changed the most (Figures 2A,B), with the return to ST facilitating them more than their counterparts who had already been advised to go to bed and get up earlier. Along the same lines, a trend difference [*F*_(5, 355)_ = 0.8, 0.05 < *p* < 0.1] between the two intervention groups was observed in midsleep on study days until T10 (earlier midsleep in the “Bright days and dark nights” group). All other sleep quality/timing variables were comparable between the two groups (Supplementary Table 2).

Significant changes in most sleep quality (i.e., fewer night awakenings) and sleep timing (i.e., less social jetlag) variables were observed in both groups over time, most likely in relation to the combination of the shift to/from DST and the course of pandemic-related events that characterized 2020 (Figures 2A–C). This is why time points were not cumulated to increase statistical power. When students were classified into four groups based on the Self-ME, their actual sleep–wake timing reflected their Self-ME at T0. Over time, the four Self-ME groups responded in a similar fashion but to a different extent to the 2020 events (Figure 2C). When sleep–wake timing was compared between commuters and on-site students at T0 (October 2019) and T6 (full lockdown in April 2020), commuters’ sleep–wake habits became more similar to those of on-site students, and their social jetlag decreased (Supplementary Figure 1).

**FIGURE 3:**
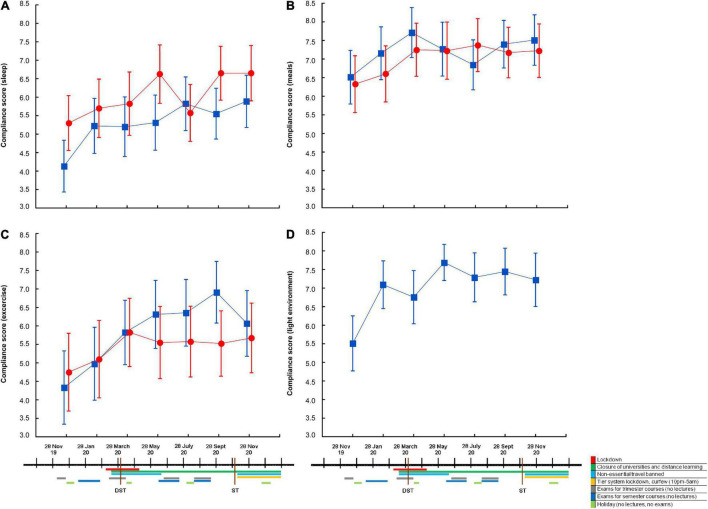
Compliance to different types of advice by circadian hygiene education group, over time, and in relation to relevant 2020 events. Self-reported compliance to advice relating to sleep **(A)**, meals **(B)**, exercise **(C)**, and light environment **(D)** in the “A regular life” (red circles; *n* = 40 at each time point) and the “Bright days and dark nights” (blue squares; *n* = 45 at each time point) groups. Significant differences between the two groups were observed in compliance to sleep advice **(A)**, which was greater in the “A regular life” group [*F*_(6, 498)_ = 6.0, *p* time < 0.0001, *F*_(1, 83)_ = 4.1, p group = 0.045, *F*_(6, 498)_ = 1.6, *p* interaction n.s.]. Compliance to all types of advice increased over time in both groups (*p* time always significant, with no further differences between groups and no interactions). Values are expressed as means ± 95% confidence intervals. n.s., not statistically significant.

Compliance completion rates decreased over time (Supplementary Table 1); 40 students completed all odd study times in the “A regular life” group, and 45 in the “Bright days and dark nights” group. Differences between the two groups were observed in compliance to sleep-related advice, which was greater in the “A regular life” group (Figure 3A). Compliance with all types of advice increased over time in both groups (Figures 3A–D). Consistent differences in compliance to different types of advice were observed in both groups [meals > sleep > exercise in the “A regular life” group; *F*_(2, 7,168)_ = 511, *p* < 0.001, all *post hoc* significant; meals and light environment > exercise > sleep in the “Bright days and dark nights” group; *F*_(3, 9,843)_ = 324, *p* < 0.001, all *post hoc* significant except for meals vs. light environment]. These relationships held true when analyzed at T1 (highest single completion rate with 920 students in the “A regular life” and 841 in the “Bright days and dark nights” groups), at complete odd time points cumulated (280 vs. 315) and at all time points cumulated (3,585 vs. 3,282) (Supplementary Table 3).

More compliant students exhibited better sleep quality on both total PSQI and on the STSQS 1–10 self-reported sleep quality score [both ANOVA and correlation analyses; i.e., PSQI vs. compliance to sleep advice: *n* = 2,188; *r*^2^ = −0.099, *p* < 0.0001], irrespective of advice group. Later chronotypes exhibited lower compliance compared with morning chronotypes [both ANOVA and correlation analyses], in most instances irrespective of advice group. In the case of compliance to sleep advice, this was also higher in the “A regular life” group, irrespective of chronotype [advice group: *F*_(1, 1,753)_ = 81, *p* < 0.0001; chronotype: *F*_(3_, _1,753)_ = 27, *p* < 0.0001; advice group * chronotype: *F*_(3_, _1,753)_ = 11, *p* not statistically significant (n.s.)].

### Academic Performance

**FIGURE 4:**
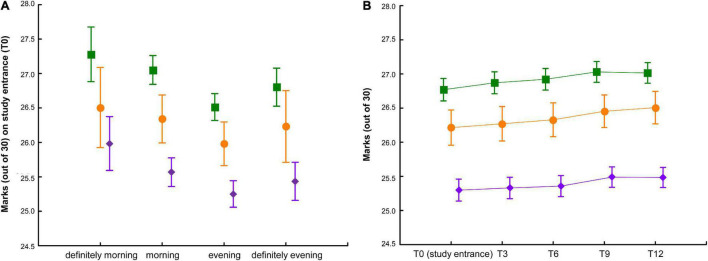
Average marks by study subject, chronotype, and over time. **(A)** Average marks (out of 30) by study subject [science and technology (S): purple diamonds; health and medical (M): orange circles; social and humanities (H): green squares] and by chronotype (definitely morning *n* = 349, morning *n* = 1,193, evening *n* = 1,390, definitely evening *n* = 647) [*F*_(3, 3,577)_ = 8.7, *p* chronotype < 0.0001, *F*_(2, 3,577)_ = 90.0, *p* study subject < 0.0001; *F*_(6, 3,577)_ = 0.3, *p* interaction n.s.] on study entrance (T0). **(B)** Average marks (out of 30) by study subject [science and technology (S, *n* = 858): purple diamonds; health and medical (M, *n* = 332): orange circles; social and humanities (H, *n* = 820): green squares] over time, aligned based on the date of joining the initiative [*F*_(4, 8,028)_ = 123.5, *p* time < 0.0001, *F*_(2, 2,007)_ = 96.3, *p* study subject < 0.0001; *F*_(8, 8,028)_ = 2.8, *p* interaction < 0.0001]. Values are expressed as means ± 95% confidence intervals. n.s., not statistically significant.

Females had better academic performance than males at all time points [i.e., 26.3 ± 2.4 (out of 30, 18 = pass) vs. 25.8 ± 2.6 on study entrance T0, *t*_(3,604)_ = 6.6, *p* < 0.0001]. Morning had better academic performance than evening chronotypes, within each study subject (Figure 4A). Students with good sleep quality (PSQI < 5) had better marks at all time points [i.e., 26.3 ± 2.4 vs. 25.9 ± 2.5 on study entrance T0, *t*_(3,604)_ = 3.8, *p* < 0.001].

As expected, the number of examinations/credits increased over time. Average marks also increased over time (Figure 4B). Students’ academic indices were comparable between the two circadian hygiene advice groups at T0 and remained so over time.

There were institutional differences in the timing of classes for M, S, and H students. Based on lectures room bookings and attendance data, M students started earlier than S students, who started earlier than H students. Further, the total lecture time was longer for M compared with S/H students. Morning examinations were scheduled earlier for M compared with S/H students, and afternoon examinations were scheduled later for H compared with M/S students (Table 3).

**TABLE 3:** Lecture and examination booking times, plus electronic attendance records, by study subject.

		Social and humanities (H)		Health and medical (M)		Science and technology (S)	
Lecture bookings	Morning start (hh:mm)	10:32 ± 01:33	*n* = 1,141	09:43 ± 01:21*	*n* = 621	10:19 ± 01:29^$#^	*n* = 2,070
	Morning end (hh:mm)	12:34 ± 01:34		12:30 ± 01:23		12:23 ± 01:34^$^	
	Afternoon start (hh:mm)	15:14 ± 01:03	*n* = 667	14:30 ± 01:01*	*n* = 387	14:52 ± 00:56^$#^	*n* = 1,036
	Afternoon end (hh:mm)	17:15 ± 01:05		17:25 ± 01:09		17:03 ± 01:03^$#^	
	Length (min)	122 ± 28	*n* = 1,808	171 ± 64*	*n* = 1,008	127 ± 47^$#^	*n* = 3,106
Examination bookings	Morning start (hh:mm)	09:50 ± 01:09	*n* = 508	09:28 ± 01:04*	*n* = 451	09:42 ± 00:58^#^	*n* = 841
	Afternoon start (hh:mm)	14:53 ± 00:48		14:32 ± 00:49*		14:34 ± 00:39^$^	
Single swipe	Start (hh:mm)	10:49 ± 02:17	*n* = 4,648	09:52 ± 02:02*	*n* = 3,783	10:33 ± 02:20^$#^	*n* = 7,062
	Length (min)	127 ± 27		184 ± 57*		131 ± 57^$#^	
Multiple swipes	First swipe in (hh:mm)	09:48 ± 01:35	*n* = 1,890	09:11 ± 01:17*	*n* = 1,969	09:52 ± 02:03^#^	*n* = 3,806
	Last swipe out (hh:mm)	15:34 ± 01:56		15:40 ± 02:11		14:53 ± 02:21^$#^	

*p < 0.001 H vs. M. ^$^p < 0.001 H vs. S. ^#^p < 0.001 M vs. S on post hoc comparisons (Bonferroni-corrected threshold: 0.05/11 = 0.0045).

When variables shown to affect sleep and/or academic performance [sex, study subject, commuting, sleep quality (continuous) and chronotype, plus age (continuous) as an adjustment factor] were included in a linear regression model, study subject (M > H > S), age (positive correlation), sleep quality (positive correlation), and sex (F > M) were associated with a higher number of examinations passed/credits acquired, whereas study subject (H > M > S), chronotype (M > E), and sleep quality (positive correlation) were associated with better marks (Supplementary Table 4).

## Discussion

Poor sleep quality was common, with more than half of the students reporting abnormal, albeit mild, sleep disruption. Although direct comparisons are not necessarily easy, as published studies on poor sleep in university students are heterogeneous for country, student populations, questionnaire/assessment tools, and/or questionnaire thresholds utilized, our results seem slightly more encouraging than those of Reis et al. (2021), who recently reported a prevalence of insomnia of 67.7% in college students in Portugal, and those of Driller et al. (2021), who reported 67% between bad and moderate sleepers among first-year health degree undergraduates in New Zealand. Despite reasonable sleep duration, social jetlag (Wittmann et al., 2006; Korman et al., 2020) was also common. All such abnormalities were less pronounced in the October 2020 compared with the October 2019 cohort, suggesting that long periods of full/partial lockdown and distance learning (thus less pressure from the “social clock”) made it easier for students to follow their inclination toward later sleep timing and to sleep better. This is supported also by the large decrease in social jetlag observed in commuters between October 2019 and April 2020 and is in line with similar, albeit mostly retrospective and unpaired observations in varying populations (Blume et al., 2020; Gao and Scullin, 2020; Korman et al., 2020; Leone et al., 2020; Cellini et al., 2021; Hallman et al., 2021; Partinen et al., 2021) and also in university students (Wright et al., 2020; Marelli et al., 2021). Most of the above studies on the effects of COVID-19–related lockdown measures and circadian/sleep variables in students and other populations were initiated during the pandemic, asking participants to provide pandemic and pre-pandemic information (the latter by recall) at a single moment in time. This methodology can be, of course, accepted but is far from ideal, because recollection of sleep–wake information (as recollection of other subjective variables) has been associated with both recall bias (Sukul et al., 2020) and summary distortion (Montagnese et al., 2009). By contrast, we were able to acquire repeat circadian/sleep information prior to and during the pandemic in a clean, longitudinal fashion, and with high time resolution. The “Bright days and dark nights” advice resulted in earlier get-up time/earlier midsleep and decreased latency between wake-up and get-up time, with no impact on sleep duration. These are desirable, relevant effects in young adults (Bjorvatn and Pallesen, 2009), and were observed within the context of a “low-intensity” intervention to promote healthy circadian behaviors in university students. One must remember that expectations on the real-life effects of educational interventions in healthy individuals are low as unsolicited advice is rarely complied with, there is an inherent resistance to changing habits (dietary advice is the most common reference in this respect; Kapur et al., 2008; Martis et al., 2018), and this is even more pronounced in healthy individuals, who have limited incentive to do so. Also, although we opted for a comprehensive baseline sleep–wake assessment, the intervention was circadian in its essence and meant to affect sleep timing rather than sleep quality variables, which is what happened until T10, which corresponded to the last time of data collection before the transition from DST to ST. The effect on sleep timing was observed despite students reporting difficulties in complying with sleep advice and may be due, in part, to them finding it easier to follow light environment advice. This type of light advice, in a population that makes large use of portable devices in the evening/early night hours for study and recreational purposes, may indeed be crucial (Gringras et al., 2015), also explaining part of the differences in the effects of the “Bright days and dark nights” and the “A regular life” advice. Being a university-based educational initiative, the study obviously lacks a placebo arm and some of the methodological rigor associated with pre-ordinated design. On the positive side, the ecological nature of the presented results provides a phenomenal amount of real-life information, which may be useful to plan similar initiatives or more formal studies in future (i.e., amount of material to fill in, amount of advice provided and its format, timing between questionnaire administration, compliance rates to different types of advice, etc.). The initial response to the initiative, as measured by standard e-mail marketing indices, compares favorably with average responses to Italian work/education-related initiatives in which candidate participants were contacted by e-mail.^2^ However, completion of questionnaires/compliance decreased considerably over time, suggesting feasibility issues with the initiative recall/refresh frequency, which may need modulating. It should be highlighted, however, that our intervention, lasting 1 year and based on monthly monitoring, was much longer and included considerably more time points than the few published similar interventions (Hershner and O’Brien, 2018; Illingworth et al., 2020; van Rijn et al., 2020; Semsarian et al., 2021). For example, Semsarian et al. (2021) report 35% participation on second contact; ours was similar, with 32% of students responding at T1 (Supplementary Table 1). In addition, lower completion rates after T0 do not necessarily imply lack of compliance with the advice received on joining the initiative. In addition, should more or less compliant students adhere to some or all the received “Bright days and dark nights” advice over long periods (i.e., it is very easy to imagine them making a habit of using the night-shift function on their portable devices), its positive effects may cumulate over time. Selection bias in relation to varying compliance levels is possible but unlikely, as students who completed all procedures at all times did not have distinctive demographic/sleep features. Further, students with later chronotypes and worse sleep quality also had slightly but significantly lower average compliance levels. Finally, while it is possible to hypothesize that the “Bright days and dark nights” advice provided to the small number of students with a definitely morning chronotype and/or a negative social jetlag may have been counterproductive, it should be highlighted that even definitely morning students had a social jetlag, which was close to 1 h, thus most likely benefiting from the suggestions received.

The published, original, and also review/meta-analytic literature on education (mostly sleep but also circadian) aimed at adolescents (Chung et al., 2017; Illingworth et al., 2020; van Rijn et al., 2020) or university students (Dietrich et al., 2016; Hershner and O’Brien, 2018; Semsarian et al., 2021) is not conclusive; it is based on shorter programs, yielding generally short-lived effects. In addition, it suggests that more consistent interventions compared with ours in terms of provision of information on the science/medical evidence behind the advice delivered tend to result in increased knowledge but not necessarily in significant changes in sleep/circadian habits (Illingworth et al., 2020; van Rijn et al., 2020). Along the same lines, and despite being appreciative of the importance of entrainment dynamics [synchronization between the endogenous and the solar clock (Roenneberg and Merrow, 2007)], our intervention was aimed at reducing the time gap between the students’ endogenous clock and the social one, because for the time being, lesson/examination times, like most social constraints, cannot be changed in the real world. So, while the advice provided might not have necessarily favored/paralleled entrainment in natural conditions, it will have hopefully helped the students functioning in their real world, with all its imposed time constraints, including lesson/examination times and DST.

The time course of sleep timing/quality between October 2019 and October 2020 suggests that the mixture of full/partial lockdown, distance learning, and the transition to/from DST all had profound and intertwining effects, with any form of relaxation of the “social clock” resulting in delayed sleep timing, longer sleep duration, and better sleep quality. These changes were always more obvious in evening types. Evening types also had worse academic performance compared with their more morning counterparts, within any study subject area. This is only partially in line with previous observations in high school students, whose performance was affected by chronotype mostly for scientific subjects (Zerbini et al., 2017).

Average marks increased over time, albeit very slightly. This might be explained by the fact that there is a selection process throughout university, with specific subjects—which may be more interesting for the students—being usually concentrated toward the end of their careers. However, it should be highlighted that the Italian system does not allow for a significant amount of choice/changes of the core curriculum, and these choices are probably of limited impact over the course of a single year, which was our analysis period. Alternative possibilities include some form of progressive familiarization with the examination system, especially for younger students, or even distance learning during 2020 affecting both students’ preparation and professors’ attitudes.

In the current study, both sleep quality and chronotype independently predicted academic performance, together with the study subject. Based on these data and the observed, institutional differences in lecture timing/duration and examinations timing between study subjects, two approaches could be envisaged, at least in Italian universities: (i) delaying science and technology timetables by 30–60 min (overall study day duration would allow to accommodate this as the last swipe out for the science and technology students was between 30 and 45 min earlier compared with the other two study subjects; Table 3); (ii) acquiring chronotype information from students (by the one-question Self-ME, which is fast and convenient), and taking these into account when designing timetables, with a view to limit pressure on evening chronotypes. This not only seems feasible and worth investigating but may also become necessary in the post-pandemic era, which will presumably be still characterized by the need to avoid large groups of students attending lectures at any given time. Thus, should lectures/examinations timetable shifts remain necessary in large universities such as ours, they should probably be better designed to maximize academic performance. Similar approaches (i.e., acquisition and use of chronotype information to design work shifts) have already proven effective in terms of sleep length in workers from major industries (Vetter et al., 2015). In addition, the advice shown to be easier to follow (i.e., advice on meal timing and light environment from “Bright days and dark nights”) could readily be provided to students when they register at the university as part of their induction package.

The main limitations of this study include: (i) the lack of a placebo arm, (ii) a decrease in adherence to completion of questionnaires/compliance over time, and (iii) the possibility that the “Bright days and dark nights” advice provided to the small number of students with a definitely morning chronotype may have been unnecessary. Despite these limitations, this large and comprehensive set of baseline data, the response and compliance to the educational initiative, the time course of sleep timing in the year of the pandemic, and the observed differences in academic performance by chronotype all underlie the importance of designing circadian-friendly university timetables.

## Data Availability Statement

The raw data supporting the conclusions of this article will be made available by the authors, without undue reservation.

## Ethics Statement

The studies involving human participants were reviewed and approved by the Azienda Ospedale – Università Padova. The patients/participants provided their written informed consent to participate in this study.

## Author Contributions

SM, LZ, CF, PF, DM, ST, DS, and RC designed the study and developed the study methods. SM, LZ, GG, CM, CI, PF, ST, DS, and RC analyzed the data. AP, DM, RR, and RV contributed to the interpretation of the data. SM and RC drafted the manuscript. All authors critically reviewed the manuscript and approved the final version.

## Conflict of Interest

PF is the Chief Executive Officer and owner of reMedia Srl, which received compensation for the development of the full-responsive website. The remaining authors declare that the research was conducted in the absence of any commercial or financial relationships that could be construed as a potential conflict of interest.

## Publisher’s Note

All claims expressed in this article are solely those of the authors and do not necessarily represent those of their affiliated organizations, or those of the publisher, the editors and the reviewers. Any product that may be evaluated in this article, or claim that may be made by its manufacturer, is not guaranteed or endorsed by the publisher.

## Footnotes

1
https://sleeprhythm-unipd.it

2
https://www.mailup.com/
